# Random and Directed Movement by Warren Root Collar Weevils (Coleoptera: Curculionidae), Relative to Size and Distance of Host Lodgepole Pine Trees

**DOI:** 10.1093/jisesa/ieaa063

**Published:** 2020-07-24

**Authors:** Sharleen L Balogh, Niklas Björklund, Dezene P W Huber, B Staffan Lindgren

**Affiliations:** 1 Natural Resources and Environmental Studies Institute, University of Northern British Columbia, Prince George, BC, Canada; 2 Department of Ecology, Swedish University of Agricultural Sciences, Uppsala, Sweden

**Keywords:** *Hylobius warreni*, host location, visual cue, random movement, Björklund funnel trap

## Abstract

*Hylobius warreni* Wood (Coleoptera: Curculionidae) is a pest of conifers, especially lodgepole pine (*Pinus contorta* var. *latifolia* Douglas ex Loudon) (Pinales: Pinaceae) in the Interior of British Columbia. The larvae feed on the roots and root collars and cause girdling damage, resulting in mortality or growth reductions. Previous research has suggested the adult weevils locate potential host trees by using random movements and vision, but likely not chemosensory cues. The purpose of this study is to determine if adult *H. warreni* respond to particular tree characteristics versus encounter potential hosts at random. Study A was a capture–mark–recapture experiment where weevils were captured on mature pine trees, while Study B was a tracking experiment within a young pine plantation. Weevils showed a preference for larger trees, and for trees that were closer to the weevil’s last known location. In Study A, weevils also avoided climbing trees in poor health, while in Study B, the weevils’ preference for taller trees increased as their distance from the weevil increased, as well as when taller trees were closer to other trees. Movement rates were similar to those observed in previous studies, were positively correlated with the average spacing of trees, and declined with time after release. This confirms previous findings that *H. warreni* may locate host trees by both vision and random movements, and that their movements are determined primarily by the size and distribution of potential host trees within their habitat.

Knowledge of insect dispersal mechanisms and patterns is critical to developing management plans for pests, as well as for modeling (Romero et al. 2010). Optimal foraging theory suggests that animals should use information from their environment in order to optimize the quantity and quality of resources acquired, for a given amount of time and energy invested (Bell 1991). This suggests that insects should use directed movements towards potential resources in order to optimize their efficiency. On the other hand, Bernays and Chapman (1994) note that there is often a very strong random element to host finding, and that some modeling approaches have shown that in certain situations, random movements may actually be more efficient than directed movements. For example, Björklund et al. (2005) found that many individuals of the pine weevil (*Hylobius abietis*) were captured in pitfall traps without any attractive stimuli, suggesting that the weevils would randomly encounter large numbers of their host pine trees in their natural environment.

The Warren root collar weevil, *Hylobius warreni* Wood (Coleoptera: Curculionidae) is a large and long-lived weevil, measuring approximately 12–15 mm in length, and living up to 5 yr as an adult (Wood 1957, Cerezke 1994). Adults are flightless and nocturnal, and use live coniferous trees as hosts for their feeding and reproduction, particularly lodgepole pine, *Pinus contorta* var. *latifolia* Douglas ex Loudon (Pinales: Pinaceae) (Cerezke 1994). They ascend trees at night to feed on bark, although this causes minimal damage to the host (Cerezke 1994). Females lay their eggs on the roots and root collars of their hosts, where the larvae develop as they feed on bark and phloem (Warren 1956, Cerezke 1994). The larval feeding can result in girdling and subsequent mortality of small trees, or growth reductions for larger trees (Warren 1956; Cerezke 1972, 1974, 1994; Schroff et al. 2006). *Hylobius warreni* has historically been a pest of only minor importance across North America, including central British Columbia (Cerezke 1994). However, the problem has increased in magnitude following the recent mountain pine beetle (*Dendroctonus ponderosae*) epidemic of the region. Reforestation efforts have created monoculture blocks of young, susceptible lodgepole pine, and weevils remaining in pine beetle-killed blocks have become concentrated on remaining pine and adjacent replanted cutblocks (Klingenberg et al. 2010a). Additionally, planted trees have been shown to be more susceptible to the effects of the weevils than naturally regenerated trees due to their smaller and more deformed roots (Robert and Lindgren 2010).

The flightless adult *H. warreni* disperse exclusively by walking. Previous studies suggest movement rates on the order of 1–2 m/d (Cerezke 1994, Machial et al. 2012a), that these movements primarily function to locate potential host trees and habitats (Cerezke 1994, Klingenberg et al. 2010b, Machial et al. 2012a), and that their movements may be closely correlated with the distribution of potential host trees within their environment. For example, Cerezke (1994) found that when weevils were captured at different trees on successive nights, the mean dispersal distance/night closely matched the mean distance between trees within the stand. In addition, observed movement rates are higher in unfavorable habitats such as those with dead trees (Klingenberg et al. 2010b), or in an open field (Machial et al. 2012b), when compared with movements in more favorable habitats with live trees. Finally, weevils that are released and not in contact with appropriate host trees tend to have movement rates that decline with time, suggesting that the weevil’s movements may function primarily to locate host trees, and then decrease once suitable resources have been located (Klingenberg et al. 2010b, Machial et al. 2012b).

Insects may seek out hosts for differing reasons, such as food, shelter, location of mates, or oviposition, depending on species, as well as on the individual’s internal state, such as hunger status or degree of sexual maturity, and external factors, such as weather or presence of predators (Laing 1937). Previous work has suggested a strong random component to *H. warreni* movements, as the weevils’ movements have been observed to be non-directional in unfavorable environments including habitats with dead trees (Klingenberg et al. 2010b) and an open field (Machial et al. 2012b). Similarly, Schroff et al. (2006) found that the spatial distribution of weevil attacks in lodgepole pine plantations appeared to be randomly distributed, rather than aggregated or clumped. However, there is also evidence that the weevils use directed movements, guided at least partially by visual cues, to locate potential hosts. Machial et al. (2012a) found that *H. warreni* is attracted to artificial tree silhouettes in the absence of olfactory stimuli, with a preference for full-tree silhouettes over partial ones, and that blinding the weevils reduced their host-finding ability. It is unclear, however, whether the weevils use any external cues to guide their movements towards trees that represent potentially more appropriate hosts, or if they move towards all tree silhouettes equally and then determine the host’s suitability once they contact it. For example, Machial et al. (2012a) did not observe an apparent preference for different colors of artificial tree silhouettes. In addition, no chemosensory component has been found with regards to the weevils’ host location. Unpublished data from B.S. Lindgren (cited in Duke and Lindgren 2006) suggests that *H. warreni* is not attracted by either α-pinene or ethanol. They also do not appear to be attracted to the volatile mixture emitted from cut pieces of host material (unpublished data), while Duke and Lindgren (2006) found no correlation between overall monoterpene content of lodgepole pine trees and attack rates by the weevils. However, there is still a possibility that *H. warreni* may use some unknown chemical cues in conjunction with visual cues. For example, research on the pine weevil (*H. abietis*) suggests that individuals can locate trees by either vision or olfactory cues alone, or by both visual and chemical cues, and the addition of a chemical attractant strengthens the visual host-finding response (Björklund et al. 2005).

Attack rates of larval *H. warreni* are higher on taller (Schroff et al. 2006) and larger diameter (Cerezke 1994) trees. Cerezke (1994) suggested that this size–attack relationship is a direct result of the additional area of healthy bark available for oviposition on larger trees. Adults of *H. warreni* also appear to ascend larger trees more frequently than smaller ones (Klingenberg et al. 2010b). However, it is uncertain if the weevils preferentially distinguish and locate larger trees when searching, or if they encounter trees of all sizes at random and then subsequently select for larger trees during their assessment of host suitability.

The use of harmonic radar technology has made it possible to track the movement of large insects such as *H. warreni* in the field. Harmonic radar was first adapted from its original purpose (location of avalanche victims) and described for use to track insects by Mascanzoni and Wallin (1986). The system works by a hand-held detector emitting a microwave beam (917 MHz), which a transponder (Schottky barrier diode attached to a metal wire antennae) attached to the insect passively reflects back at twice the frequency (Machial et al. 2012b). Since it is a passive system, the transponder does not need an attached energy source like more traditional radio transmitters, and thus can be light and small enough to use with insects (Lövei et al. 1997, Reynolds and Riley 2002). Harmonic radar has also been shown to yield much higher recapture rates than mass capture methods such as pitfall traps or funnel traps, with recapture rates up to 94% (Williams et al. 2004, Klingenberg et al. 2010b, Machial et al. 2012b). It also compares favorably to mass trapping since it does not interrupt the insect’s movements during the study (Charrier et al. 1997, Vinatier et al. 2010). In addition, the invention of the Björklund funnel trap (Björklund 2009), which intercepts weevils as they ascend trees for feeding, has made it possible to more easily study the host tree selection of adult weevils.

This study utilizes the above tracking and trapping methods to assess the movements and host selection behavior of adult *H. warreni* in two types of potentially suitable habitat: a mature artificially regenerated lodgepole pine stand and a young naturally regenerated stand, to determine: 1) if *H. warreni* dispersal is predominantly directional or random, and 2) if *H. warreni* is preferentially attracted to specific trees while locating new hosts, and, if they are, if that preference can be explained by specific characteristics such as physical traits of the trees or spatial characteristics such as relative distance to those trees.

## Methods

### Study A: Movement Patterns and Rates

This study was conducted in the spring of 2006, during the testing of the newly designed Björklund funnel trap (Björklund 2009). The study site was an artificially regenerated lodgepole pine stand of approximately 1 ha, within the city limits of Prince George, BC (53°55′N, 122°49′W). We designated half of the stand (~0.455 ha) as the study area, recorded x–y coordinates and stem diameter at the root collar of all trees, and we noted any discoloration of the needles, and whether or not the tree was leaning. We attached Björklund funnel traps to all 182 trees in the study area. The traps were constructed of a semi-circle of asphalt-saturated kraft paper (Vaporex 400S, Building Products of Canada Corporation, LaSalle, QC) with one side coated with a strip of a fluoropolymer resin (AD1070, AGC Chemicals Americas, Inc., Bayonne, NJ) and then wrapped around the bole of the trees, as per the procedures outlined in Björklund (2009). The trap functions due to the adult weevil’s climbing and descending behavior during the night, as individuals are able to climb up the outside of the trap but then fall into the funnel. This trap design has been shown to be effective at capturing individuals of *H. warreni* during subsequent studies (Klingenberg et al. 2010b; Machial et al. 2012a, b).

We installed the first 100 traps on May 26th, a further 18 on May 27th, 13 on May 28th and the final 51 on May 29th 2006. We checked the traps each morning for any captures of *H. warreni* adults, which we gave a unique identifier by marking them with liquid paper and an individual numeric mark, and we recorded any recaptured individuals. We released all captured weevils immediately at the base of the tree where they were caught. We ran the experiment for 13 d, ending on June 11th 2006. For further details on the experimental methods, see Björklund (2009). A preliminary analysis of the data was limited to showing that weevils preferred larger diameter trees, were captured on 10–38% of the trees on any one day, and did not decline over the period of trapping (Klingenberg et al. 2010b).

### Study B: Movement, Host Location, and Selection

#### Capture and Storage of Specimens

We captured a total of 466 adult *H. warreni* in lodgepole pine stands in the Prince George, BC area, during the spring and summer of 2013 and 2014 (181 individuals in 2013 and 265 individuals in 2014). We used a combination of Björklund funnel traps (Björklund 2009) and manual searching to capture weevils. We constructed funnel traps as described above for Study A, using asphalt-saturated kraft paper (Vaporex 400S), coated with fluoropolymer resin (Teflon PTFE DISP 30, DuPont, Wilmington, DE). Manual searching involved pulling back the duff layer around the base of lodgepole pine trees, and locating weevils hiding either under the duff or on or around the root collar of the tree.

In 2013, the majority of the weevils were obtained by manual searching. Björklund funnel traps set up in seven stands yielded few weevils, likely due to interference of the trap by forest tent caterpillars (*Malacosoma disstria*) and their silk. In 2014, we set up 270 traps in early spring (April–May) in a single stand at the Prince George Tree Improvement Station (PGTIS), with high rates of trapping success. Thus, most of the weevils captured in 2014 were captured by the use of the Björklund funnel traps in this single stand. On return to the laboratory, we determined the sex of captured insects based on the identification of two external characters on the weevils (Öhrn et al. 2008). When both characters matched, this method has been shown to be 97% effective (Öhrn et al. 2008), in comparison to identification of sex by dissection.

We stored captured weevils at room temperature in the dark in the laboratory at the University of Northern British Columbia (2013), or in a low-temperature growth chamber (B.O.D. Low-temperature incubator, VWR International, Radnor, PA) at 6–7°C and ~50–60% humidity (2014). Until used for experiments, we kept between one and six weevils in 0.95-liter plastic food storage containers (TakeAlong Rectangles, Rubbermaid, Atlanta, GA) with mesh inserts in the lids to allow ventilation. We stored males and females separately. We provided water (moistened paper towels) and food (freshly cut lodgepole pine branches) ad libitum, and checked containers for food and water requirements at least twice per week.

#### Tagging and Marking of Weevils

**Fig. 1.**
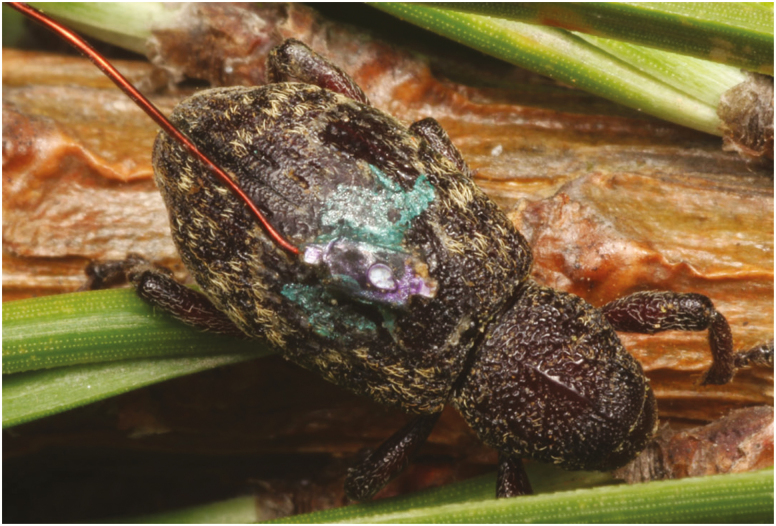
Attachment of transponder to the elytra of an individual of *Hylobius warreni*, showing the three-color marking system on the diodes. Photo by BSL.

We used harmonic radar technology for tracking insects in the field. We constructed harmonic radar transponders of a 41-mm-length × 0.5-mm-diameter straight copper wire antenna soldered to one pole of a Schottky diode. We selected the antennal length based initially on a previous study with *H. warreni* (Machial et al. 2012b), which used a 50-mm antenna, and found adequate detection distance (1–2 m) based on the movement rates of the species, with apparently minimal influence on movement or behavior. After preliminary testing, we shortened the antennal length to 41 mm, or 1/8 of the 917 MHz wavelength emitted by the detector. Reflection of the antenna should be maximal at fractions of the emitted wavelength (O’Neal et al. 2004), and laboratory testing indicated a similar detection distance of 1–2 m using the shorter 41 mm length (S.L.B., personal observation). We attached transponders to the elytra of the weevils with cyanoacrylate glue (Instant Krazy Glue, gel formula, Elmer’s Products, Inc., Westerville, OH), which provides a strong, durable bond and is nontoxic to insects (Boiteau et al. 2009). In addition, we gave each weevil a unique three-color code by marking the exposed three sides of the diodes of the transponders with metallic pens (The Write Dudes Infinity Metallic Permanent Marker, MEGA Brands, Inc., St-Laurent, QC; Fig. 1).

#### Study Site

We conducted all tracking experiments in a naturally regenerated stand of approximately 8 yr of age at the PGTIS (53°46′N, 122°43′W). The stand was primarily lodgepole pine (~90%) of about 1–2 m in height, with smaller contributions of similarly sized interior hybrid spruce (*Picea engelmannii × glauca*), trembling aspen (*Populus tremuloides*), paper birch (*Betula papyrifera*), larch (*Larix* sp.), and willow (*Salix* spp.) The site had low incidence of previous weevil attack, as indicated by external physical characteristics such as leaning trees or red needles on branch tips (McCulloch et al. 2009). In addition, the stand had low ground cover in order to ensure that weevils could be easily relocated, although there was some ground vegetation, including bearberry (*Arctostaphylos uva-ursi*), white Dutch clover (*Trifolium repens*), and various grasses.

We established fifteen 5-m × 5-m plots within the PGTIS stand in 2013, with plots chosen so that the center was in an open area without trees, surrounded on most or all sides by trees of differing sizes. This arrangement was chosen so that the weevils were presented with a choice of tree sizes in various directions at an appropriate distance, based on the species’ previously observed movement rates of 1–2 m/d (Cerezke 1994, Machial et al. 2012b). For site photographs and the arrangement of trees and plots within the site, see Supp Fig. B.1.1, B.1.2, and B.1.3 (online only). Plots were oriented in North–South and East–West directions, and the corners and center were marked by plastic tags inserted into the ground (Rapiclip 15-cm plant labels, Luster Leaf Products, Inc., Woodstock, IL) with surveyor’s flagging tape tied to them. We numbered and marked all trees >10 mm diameter at the root collar with plastic survey discs. This size minimum was selected to give a buffer from the 20 mm minimum size required for oviposition of *H. warreni* based on previous literature (McCulloch et al. 2009). For all numbered trees, we recorded species, stem diameter at the root collar, total height, crown diameter, and the tree’s position relative to the plot center, using a measuring tape and compass bearing.

#### Tracking

We released a total of 115 weevils in nine separate trials: 70 weevils in six trials in 2013 and 45 weevils in three trials in 2014 (Table 1). For each trial, we released 9–15 weevils in the evening (at about 1900 h), with each weevil released individually at the center of a single plot. Male and female weevils were released alternately when possible. However, transponders would sometimes become detached before or during transport to the site, resulting in the release of unequal numbers of males and females, and in turn necessitating consecutive releases of weevils of the same sex. Weevils were released oriented randomly in N, NE, E, SE, S, SW, W, or NW directions, with the direction of release determined by a random number generator. The weevil was placed so that the center marker was immediately to the right and towards the rear of the weevil, when the observer was positioned directly behind it and facing in the same direction. Previous studies have indicated that other beetle species differ in both direction and rates of movement when hungry versus when fed (Wallin 1991, Wallin and Ekbom 1994, Szyszko et al. 2004). The types of changes vary with species, but some studies have shown beetles exhibit directed movements toward food when hungry, and random movements when satiated (e.g., Wallin 1991). Therefore, we conducted some initial trials in 2013 with weevils fasted for 24 h. However, due to high levels of mortality during these initial trials, and the concern that fasting of the weevils may have been a contributing factor, we conducted later trials in 2013 and all trials in 2014 with weevils that were provided with food until approximately 1 h before release.

**Table 1.** Host location trials of *Hylobius warreni* tracked by harmonic radar in a lodgepole pine stand in the summers of 2013 and 2014

Trial	Release date	Trial length (hours)	Number of relocations	Feeding status	Number of replicates, total (M/F)
1	9 July 2013	63	7	Fasted	10 (5/5)
2	24 July 2013	43	3	Fasted	15 (7/8)
3	29 July 2013	88	5	Fed	14 (8/6)
4	6 Aug. 2013	40	3	Fasted	9 (5/4)
5	12 Aug. 2013	40	3	Fed	10 (4/6)
6	14 Aug. 2013	40	3	Fed	12 (7/5)
7	14 May 2014	40	3	Fed	15 (8/7)
8	11 June 2014	40	3	Fed	15 (5/10)
9	25 June 2014	40	3	Fed	15 (7/8)

We used a RECCO Detector (R9, RECCO AB, Lidingö, Sweden) to detect tagged insects in the field. When a signal indicating the presence of a nearby transponder was found, the operator located the point of the strongest signal, switched off the detector, and began a manual search, as per the procedures of Machial et al. (2012b). Once a weevil was found, its location was marked with a numbered plastic tag (Rapiclip 15-cm plant labels), placed so the weevil was on the right (as above). Care was taken to minimize disturbances to the weevils, but grass or other vegetation was carefully moved if necessary. If it was not possible to visually locate a weevil without unnecessary disturbance, and it did not appear to have moved based on its last known position and the location of strongest signal from the harmonic radar detector, it was assumed to be in the same position. At the conclusion of each trial, we collected and removed all weevils from the study area. Weevils were not re-used for subsequent trials. We determined the positions of all marked weevils with a measuring tape and compass bearing, measuring from the position of release (plot center) to the weevil’s location. We also took measurements in the same manner from the weevil’s location to the nearest tree.

In order to ensure that we were identifying weevils correctly as male or female via external characters, the sexes of a total of 50 weevils were determined by dissection. Dissections confirmed a total accuracy rate of 92%. Therefore, we classified any weevils which were unidentified by dissection as male or female via their external characters only.

### Statistical Analysis

We performed all analyses using R version 3.0.2 (R Core Team 2013). Unless otherwise noted, results are reported as means ± SE.

#### Study A: Movement Patterns and Rates

In order to keep consistency between capture periods, we only considered weevil captures during the period when all traps were in place in the analysis (May 30th–June 11th).

##### Movement Rates of Weevils and Spatial Characteristics of Trees

We estimated overall observed displacements over the study period for all weevils that were captured more than once. Since the weevils may not have moved in straight lines between observations, this should correspond to the minimum overall movement rates of the weevils. We determined movement distances directly from the x–y coordinates of the trees where they were captured, and measured movements as the linear distance between trees for consecutive captures, or zero if they were captured at the same tree. For each weevil, we divided the total distance it was observed to travel over the study period by the number of days between its first and last capture, to determine its individual mean movement rate. In addition, we estimated single-night dispersal distances for all weevils that were captured at different trees on consecutive days. We then combined data from all weevils to calculate descriptive statistics for both overall movement rates and single-night movement distances.

**Fig. 2.**
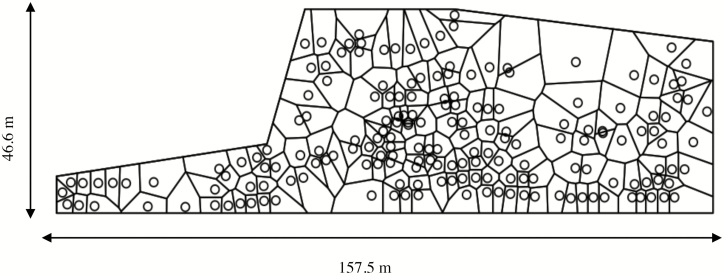
Voronoi polygons constructed by a Dirichlet tessellation used to determine neighboring trees and the area potentially available (APA) for each tree in a site used for a capture–mark–recapture study of *Hylobius warreni* in 2006. The polygonal window around the triangulation was +1.4 m from the approximate outer edge of the stand, which are represented in the figure by circles. The figure was created using the ‘spatstat’ package in R (Baddeley and Turner 2005).

We based measures of the spatial characteristics of the stand on those presented by Cerezke (1994), including both distance to nearest neighbor tree, and the average distance between trees. We calculated the linear minimum distance to the first nearest neighbor to each tree using the ‘FNN’ package in R (Beygelzimer et al. 2013), while we determined the mean distance between trees by the calculation of the average linear distance from each tree to all of its neighbor trees. We identified neighboring trees by using a Dirichlet tessellation, a method that involves the construction of ‘Voronoi polygons’ around each point in a plane. The polygons are constructed by the placement of each side of the polygon at the midpoint between the tree and its nearest neighbor in a given direction, thus perpendicularly bisecting the distance between the two points (Maclauchlan and Borden 1996). We considered neighbor trees as those that shared a side of their respective Voronoi polygons. In addition to identifying neighbor trees, the Dirichlet tessellation also gives a measure of the area potentially available (APA) for each tree, described as the area of influence of that tree in the site (Maclauchlan and Borden 1996). We constructed the polygons using the R package ‘spatstat’ (Baddeley and Turner 2005). The window surrounding the tessellation was set as an irregular polygon, with the outermost boundaries set at 1.4 m (half of the mean distance from each tree to its nearest neighbor) from the approximate outer edge of the stand (Fig. 2).

##### Effect of Physical and Spatial Characteristics of Trees on Total Number of Captures

We examined the effect of individual physical and spatial predictor parameters on the total number of captures on each tree by using a best-fit linear regression model. Parameters considered for inclusion in the model were ‘APA’, the area potentially available calculated from the Dirichlet tessellation; ‘nearest neighbor’, the distance to the first nearest neighbor tree, calculated as above for the movement rate data; ‘color’, a binary factor, representing either green or discolored yellow/red needles; ‘leaning’, whether or not the tree was visibly leaning; and ‘diameter’, the diameter of each tree at the root collar, since a preliminary analysis of this data (Klingenberg et al. 2010b), showed that tree diameter was positively related to capture frequencies. We also considered any potential two-way interactions between terms in the regression model. The continuous independent variables were centered around their respective means before analysis. The dependent variable was the total number of *H. warreni* captures on the tree, summed over the entire sampling period. In order to satisfy the assumptions of normality of residuals and homogeneity of variances, the dependent variable (total number of captures) was ln(x + 1) transformed. In addition, the APA data were square root transformed to satisfy the assumption of linearity. We selected the best-fit model on the basis of the lowest Akaike Information Criterion (AIC) value, beginning with the full model without any interaction terms, and then using a combination of forward addition and backwards subtraction with the ‘step’ function in R.

#### Study B: Movement, Host Location, and Selection

For the purposes of analysis, we considered each individual weevil released at the center of a plot as a replicate. A replicate was considered to be successful if the weevil was located alive at least twice after its release and was observed to have moved a total distance of more than 30 cm from the plot center. We established this final criterion since we often observed weevils simply burrowing down next to the plastic tag where they were released, apparently seeking shelter, and remaining there for the duration of the trial. We selected the distance of 30 cm arbitrarily, but it seemed reasonably conservative, since it represented less than one third of the distance from the nearest tree to the center in any of the plots (98 cm). We excluded from analyses any unsuccessful replicates in which the weevil was unable to be located more than once, died before it was able to be relocated twice, or moved ≤30 cm. Due to a high rate of unsuccessful replicates during some of the trials, we pooled data from all plots and trials for the analyses.

##### Movement Rates

We calculated movement rates from all successful trials, excluding the first hour of data (to allow for a settling time after handling), and until the second morning of observations following release. These data represented two distinct periods of movement: the first night (from ~2000 h to 1100 h the following morning) and the next 24 h (from ~1100 h to 1100 h the next day). Only individuals for which all observations were recorded for the given time interval (i.e., no missing data points, losses, or deaths) were used. We compared movement rates for the first night of observations (move 1) and the second night (move 2) by a paired t-test, with each pair representing the movements of a single individual. In addition, we compared movement rates (combined over both time intervals) between males and female weevils by Welch’s independent samples t-test.

##### Directionality

We assessed directionality of movement following release by the compass bearing direction that the weevil had moved for its first move of >30 cm. First, we assessed overall directions of initial movement by pooling all observations from all trials and plots and comparing these to a null hypothesis of no consistent movement direction by the Rayleigh test. This test compares the observed directions of movements of the weevils to a uniform distribution, where there is no significant mean direction (Batschelet 1981). Second, we compared the direction of a weevil’s first movement to the direction that the weevil was facing on its release, the direction of the largest tree in the plot, and the direction of the closest tree to the plot center by separate circular correlation tests. The circular correlation test calculates a correlation coefficient similar to that of Pearson’s product moment correlation, except that it computes the sine of the difference between the observation and the mean instead of only the difference (Agostinelli and Lund 2013). For the correlation of the direction of the first move and that of the nearest tree, we omitted the five observations from plot three since there were two trees equally near to the plot center, which were located in opposing directions. Finally, in all replicates where there were two distinct non-zero moves, and in which the weevil changed direction between its first and second moves, we summed and compared the total number left and right turns to an expected frequency of 50% left and 50% right by a chi-squared goodness-of-fit test. The Rayleigh test and circular correlation tests were computed using the ‘CircStats’ (Lund and Agostinelli 2012) and ‘circular’ (Agostinelli and Lund 2013) packages in R.

##### Selection of Trees

A tree was considered to have been ‘selected’ by a weevil if a weevil was found anywhere underneath the crown of that tree. In the cases where weevils selected more than one tree during their replicate, we only considered the tree that was selected first. We used a mixed-effects logistics regression to analyze the weevil’s selection of trees. The dependent variable was a binary variable, representing whether the tree was ever ‘selected’ by weevils, or was ‘not selected’, indicating if a weevil was ever found underneath it in any trial. We considered the following parameters as fixed effect independent variables in the model: ‘height’ (total tree height, from base to tip, used as a measure of tree size), ‘distance’ (linear distance of the tree from the plot center), ‘nearest neighbor’ (linear distance from the tree to its closest neighboring tree), as well as any potential two-way interactions between these effects. The random effect was plot. We centered fixed effects parameters around their respective means prior to analysis. The best-fit model was selected on the basis of the lowest Akaike’s Information Criterion (AIC) value, by a combination of forward addition and backwards elimination of both main effects and first order (two-way) interaction effects, while the overall significance of the fitted model was assessed by a chi-squared test, comparing the best-fit model with the null model containing only an intercept. We back-transformed the resulting logistic regression equation to calculate odds ratios (ORs) for the coefficients, and estimated 95% CIs for ORs by a normal approximation of SEs. The model was fit using the ‘lme4’ package in R (Bates et al. 2014).

## Results

### Study A: Movement Patterns and Rates

#### Movement Rates of Weevils and Spatial Characteristics of Trees

**Fig. 3.**
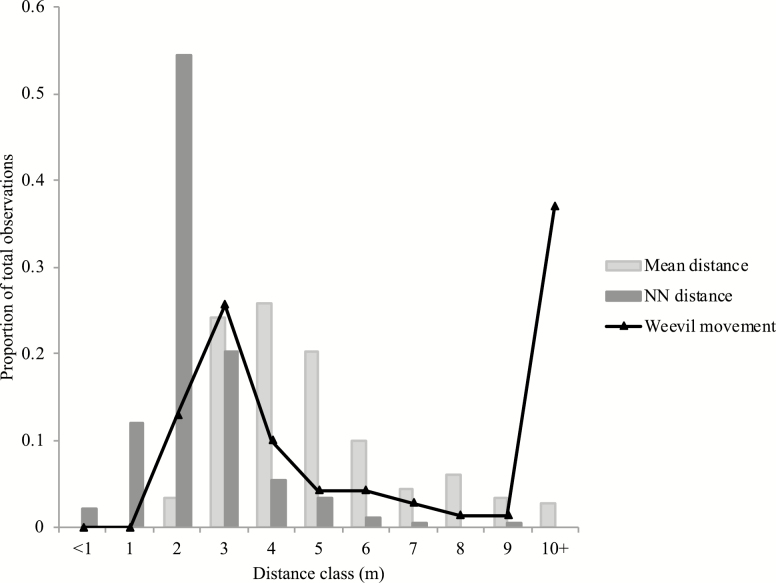
Movement distances per night of *Hylobius warreni*, when individuals were captured on consecutive days at different trees (‘Weevil movement’) in a lodgepole pine stand, compared to both the mean distances between trees (‘Mean distance’) and distance to nearest neighbor trees (‘NN distance’) within the stand. Data were collected during a capture–mark recapture experiment in the spring of 2006.

The overall average movement rate for all weevils, including days in which the weevils did not move, was 3.43 ± 0.30 m/d, with a slightly slower median movement rate of 2.33 m/d (N = 211). The average single-move length between consecutive captures of the same weevil at different trees was 14.42 ± 2.16 m, although the median single-move length was much lower at 5.07 m (N = 70) as there were several weevils that moved extremely long distances between captures. When weevils were captured at different trees on consecutive nights, both their mean and median movement distance corresponded more closely with the mean distance between trees than with nearest neighbor distances (Table 2). In addition, the distribution of the weevil movements more closely matched the distribution of the mean distance between trees, when compared with the distance to the nearest neighbor (Fig. 3).

**Table 2.** Descriptive statistics of single-night movement distances of individuals of *Hylobius warreni*, when captured on consecutive mornings at different trees in a lodgepole pine stand (‘Weevil movement’) to the mean distance between trees (‘Mean distance’) and the distance to the nearest neighbor tree (‘NN distance’) during a capture–mark–recapture experiment in the spring of 2006

	Mean distance (m)	NN distance (m)	Weevil movement (m)
Mean ± SE	5.32 ± 0.14	2.84 ± 0.087	14.42 ± 2.16
Median	4.86	2.69	5.07
*N*	182	182	70

#### Effect of Physical and Spatial Characteristics of Trees on Captures

**Fig. 4.**
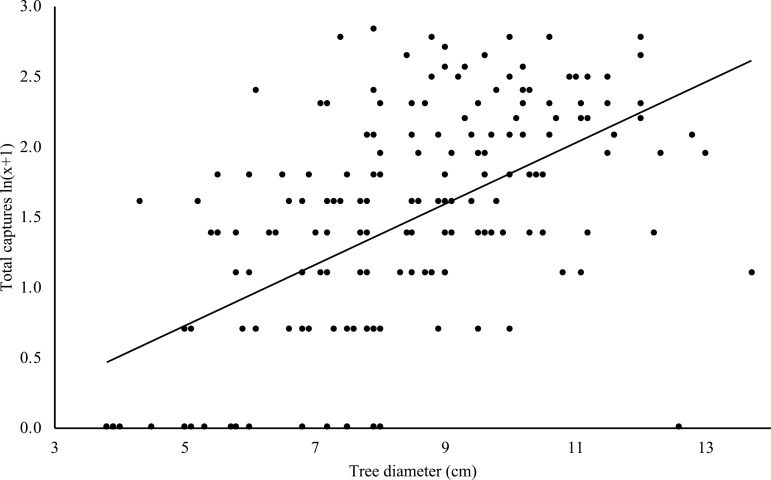
Relationship between the diameter of trees in a lodgepole pine stand, and the total number of captures of individuals of *Hylobius warreni* on individual trees over a 12-d period in the spring of 2006.

**Fig. 5.**
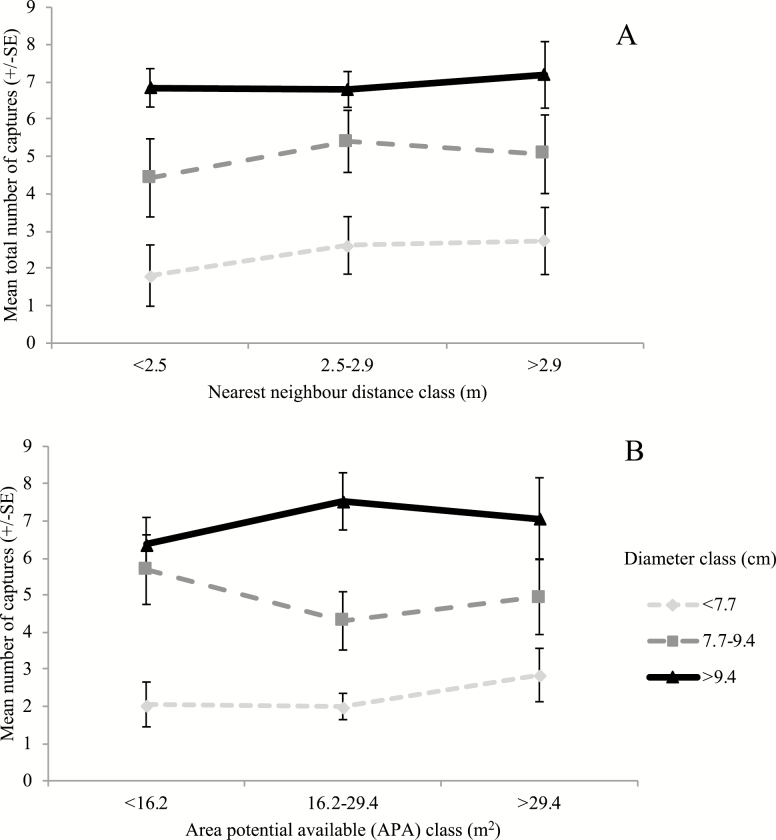
Effect of the interaction of (A) tree diameter and distance to nearest neighbor tree, and (B) tree diameter and area potential available (APA), on the total number of captures of *Hylobius warreni* for that tree, in a lodgepole pine stand, over a 12-d period in the spring of 2006.

A total of 863 captures were made by the 182 traps (many weevils were trapped more than once), with a mean capture rate of 4.74 ± 0.30 weevils per trap. There was a large amount of variability between the capture rates of the traps, as the maximum number of weevils trapped on a single tree was 16, while there were 24 trees on which no weevils were trapped. The best-fit model, selected on the basis of AIC value (Suppl Table A.1.1 [online only]), was highly significant (F_(8,173)_ = 16.36, P < 0.001), offered reasonable explanatory power (R2 = 0.43), and contained a number of parameters and interaction effects (Table 3). Of those parameters, diameter had a significant positive effect (Fig. 4), while red or yellow discolored needles had a significant negative effect (1.60 ± 0.81 captures per tree) compared to trees with green needles (4.83 ± 0.30 captures per tree). There were also significant negative interactions between diameter and distance to nearest neighbor (Fig. 5A), as well as diameter and the area surrounding each tree (APA) (Fig. 5B), on the total number of captures of each tree.

**Table 3.** Results of the best-fit linear regression equation of spatial and physical parameters on the total number of captures of *Hylobius warreni* individuals of trees in a lodgepole pine stand, selected on the basis of Akaike Information Criterion value

Parameter	Estimate	SE	*t*	*P* (>|*t*|)
**Intercept**	**1.54**	**0.048**	**31.84**	**<0.001**
**Diameter**	**0.23**	**0.023**	**9.91**	**<0.001**
APA	−0.044	0.036	−1.23	0.22
NN	−0.028	0.056	−0.51	0.61
**Color**	**−0.62**	**0.27**	**−2.31**	**0.02**
Leaning	−0.38	0.40	−0.95	0.34
NN*Leaning	−0.26	0.18	−1.42	0.16
Diameter*APA	**−0.037**	**0.016**	**−2.30**	**0.02**
Diameter*NN	−**0.062**	**0.027**	**−2.30**	**0.02**

Diameter = diameter of each tree; APA = area potentially available for each tree, square root transformed; Nearest Neighbor (NN) = distance to nearest neighbor tree; Color = a factor indicating if the needles were discolored either yellow or red; Leaning = a factor indicating if the tree was visibly leaning. The dependent variable (number of captures) was ln(*x* + 1) transformed. Statistically significant effects are highlighted in bold text.

### Study B: Movement, Host Location, and Selection

#### Tracking Success

Fifty-nine of the 115 replicates were considered successful (51%). Of those, 52% of the male weevils released resulted in successful replicates, while 51% of the female replicates were successful (Table 4). A chi-squared goodness-of-fit test of the successful replicates showed no difference from an equal likelihood of success for each sex (χ2_(1)_ = 0, N = 59, P = 1). Of the 56 replicates which were not successful, 25 were unsuccessful because the weevil was lost before it could be relocated twice, 17 were observed to have died before they could be relocated twice, and 14 were unsuccessful only because the weevil did not move more than 30 cm in total from the plot center. A replicate was sometimes considered a failure by more than one criterion. For example, most of the weevils that were found dead also had not moved more than 30 cm from their point of release. A total of 182 weevil positions were recorded among the successful replicates, in which the weevil was able to be located and was also alive. Of these 182 positions, the weevil had moved from its past location by at least 5 cm 132 times, or 73% of the time.

**Table 4.** Total and successful replicates of *Hylobius warreni* releases during a series of harmonic radar tracking experiments during the summers of 2013 and 2014

Trial	Release date	Total replicates		Successful replicates	
		Male	Female	Male	Female
1	9 July 2013	5	5	2	4
2	24 July 2013	7	8	1	1
3	29 July 2013	8	6	3	2
4	6 Aug. 2013	5	4	2	2
5	12 Aug. 2013	4	6	3	5
6	14 Aug. 2013	7	5	5	2
7	14 May 2014	8	7	6	4
8	11 June 2014	5	10	2	5
9	25 June 2014	7	8	5	5
Total		56	59	29	30

A replicate was considered successful if the weevil was located alive at least twice and had moved more than 30 cm from the plot center.

#### Movement Rates

The average movement observed over the 39 h of observations (40 h excluding the first hour) was 150 ± 20 cm, which represented a movement rate of 3.8 ± 0.5 cm/h, or 92 ± 12 cm/d. The longest movement observed over the 39 h by an individual weevil was by a male weevil, who moved a total of 626 cm. Movement rates (cm/h) for the first night (first move, 6.4 ±1.0 cm/h) were significantly larger than those for the second night (second move, 2.2 ± 0.5 cm/h) (t_(40)_ = 3.88, P < 0.001). There was no significant difference between the movement rates of male (6.8 ±1.2 cm/h) versus female (8.1 ±1.7 cm/h) weevils (t_(36.6)_ = −1.05).

#### Directionality

**Fig. 6.**
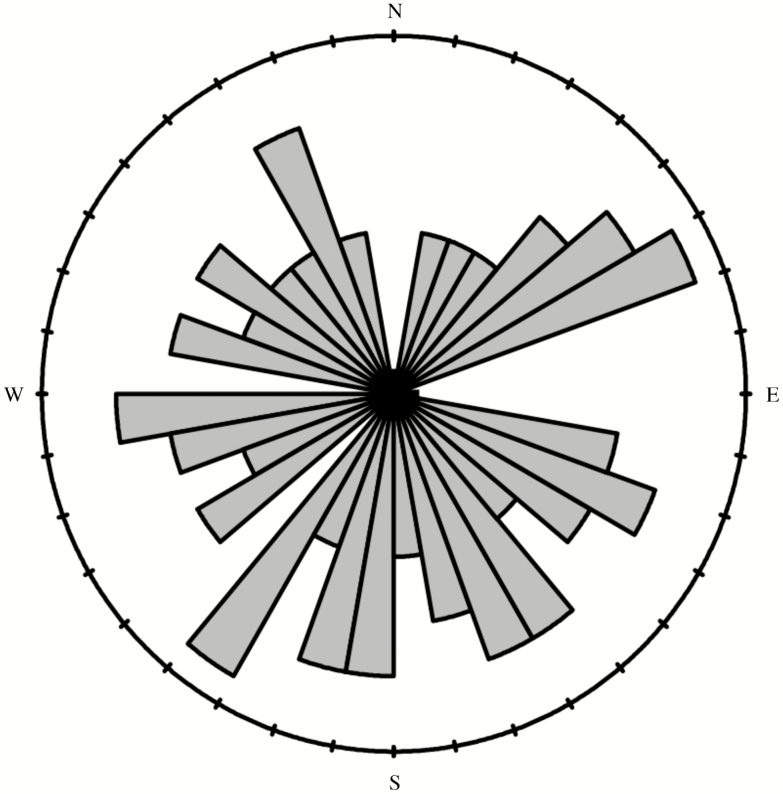
Circular histogram of frequencies of directions individuals of *Hylobius warreni* moved for their first move >30 cm in a series of harmonic radar tracking experiments during the summers of 2013 and 2014. Each bar represents 10 degrees of arc. The distribution was not found to deviate from randomness by the Rayleigh test ($\bar{r}$ = 0.15, *P* = 0.24). *N* = 59.

The Rayleigh test did not suggest that there was a consistent mean direction of the first move > 30 cm (r = 0.15, P = 0.24) (Fig. 6). In addition, there was no significant correlation between the direction of the first move >30 cm and the direction of release (r = 0.0067, P = 0.96), the direction of the first move and the largest tree in the plot (r = 0.14, P = 0.25), and the direction of the first move and the closest tree to the plot center (r = −0.058, P = 0.67). Finally, there was no apparent preference for left or right turns between successive moves, as determined by the turn angles between first and second distinct moves, as 12 of 27 weevils turned right while 15 of 27 weevils turned left (χ 2_(1)_ = 0.0186, N = 27, P = 0.89).

#### Selection of Trees

**Fig. 7.**
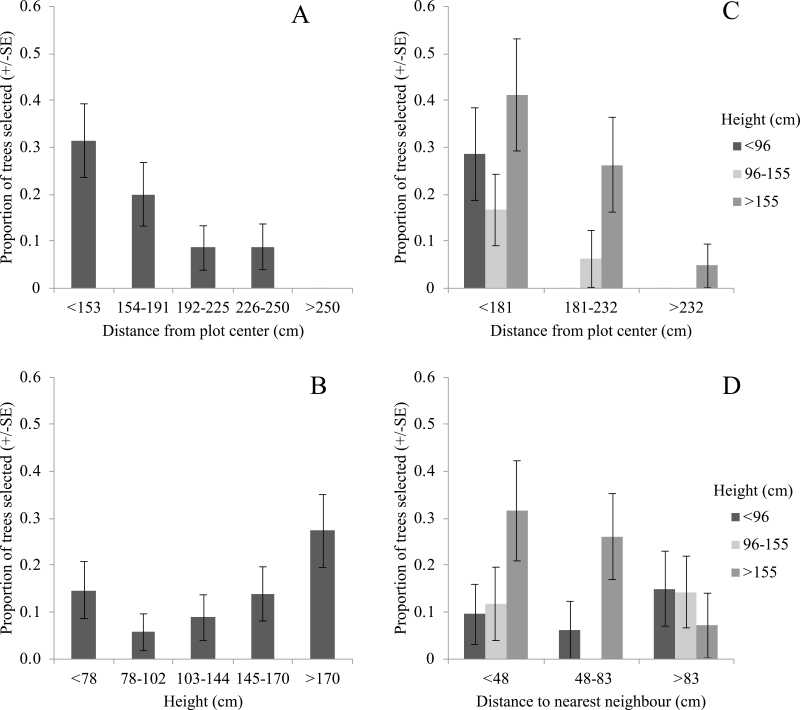
The effect of (A) distance of tree from plot center, (B) tree height, (C) interaction between tree height and distance of that tree from plot center, and (D) interaction between tree height and distance to nearest neighbor tree, on the proportion of trees selected by individuals of *Hylobius warreni* in a lodgepole pine stand during a series of harmonic radar tracking experiments in the summer of 2013 and 2014. N = 172.

There were 24 trees in 11 plots that were ‘selected’ and 148 trees that were ‘not selected’ by weevils. Of these 24 trees, 19 were selected by only a single weevil, 4 were selected twice, and 1 was selected three times. The best-fit model for the selection of trees by weevils contained the fixed effects of height, distance from plot center, and nearest neighbor, as well as the interactions of height with distance, and height with nearest neighbor distance, along with the random effect of plot (Supp Table B.2.1 [online only]) The overall model was significantly different from the null model (χ2_(5)_ = 38.65, P < 0.001). ORs and associated CIs of the resulting model are shown in Table 5. This model suggested that weevils were more likely to select trees that were closer to the plot center (Fig. 7A) and taller (Fig. 7B), and that the effect of height increased as trees were located further from the plot center (Fig. 7C). In addition, the interaction of height and nearest neighbor suggested that taller trees were more likely to be selected if they were closer to their nearest neighbor, but that smaller trees were not (Fig. 7D).

**Table 5.** Results of the best-fit mixed-effects logistic regression model for *Hylobius warreni* selection of trees from a harmonic radar tracking experiment

	Estimate	SE	*z* value	*P* (>|*z*|)	OR	Upper 95% CI	Lower 95% CI
**Intercept**	**−3.051**	**0.50**	**−6.16**	**<0.001**	**0.047**	**0.018**	**0.125**
**Distance**	**−0.032**	**0.0074**	**−4.28**	**<0.001**	**0.97**	**0.96**	**0.98**
**Height**	**0.020**	**0.0077**	**2.59**	**0.010**	**1.02**	**1.00**	**1.04**
NN	**−**0.0023	0.0057	**−**0.41	0.681	1.00	0.99	1.01
Height*NN	**−0.00030**	**0.00013**	**−2.27**	**0.023**	**1.00**	**0.99**	**0.99**
Distance*Height	0.00021	0.00012	1.82	0.069	1.00	1.00	1.00

Distance = distance of tree from the plot center; Height = total tree height; NN= distance from the tree to its nearest neighbor tree. Statistically significant effects have been highlighted in bold text.

## Discussion

Mean overall movement rates of adult H. warreni were estimated at 3.4 m/d in Study A, and <1 m/d in Study B. These rates approximate the range of previous studies, which had suggested movement rates for this species range between <1 m/d (Machial et a. 2012b) and 1–2 m/d (Cerezke 1994, Klingenberg et al. 2010b). In addition, the results of both studies support the hypothesis of Cerezke (1994) that it is primarily the stand characteristics that determine movement rates in this species, not an inherent biological limitation. In Study A, the average movement distance between trees during a single night corresponded more closely to the average distance between trees, rather than to the distance to the nearest neighbor tree, while in Study B, the close spacing of the trees as a result of their young age and small size, as well as the drought conditions during the experimental period, may have led to the slower observed movement rates. Evidence from other insect species supports this hypothesis. For example, Schneider (2003) found that when looking at butterfly (Maniola jurtina L.) movement rates in different capture–mark–recapture studies, there was a linear relationship between the scale (area) used in the study and the mean movement distance, indicating that the observed movement rates were determined more strongly by the study and site characteristics than by an inherent characteristic of the species.

Movement rates during Study B were observed to decline with time, as the first consecutive move was consistently longer than the second move by the same weevil, supporting the results of Machial et al. (2012b) and Klingenberg et al. (2010b) who also found that H. warreni movement rates declined with time after release. This suggests that once the weevils are able to locate suitable habitat they may tend to remain there, furthering the evidence that the location of appropriate habitats, such as host trees and shelter, may be the primary driving force behind their movements. In addition, there was no significant difference between male and female movement rates, suggesting that reproduction may not be a driving factor in the weevils’ movements [e.g., searching males would exhibit the majority of the movements, while reproductively mature females would remain stationary at suitable oviposition sites, as Williams et al. (2004) observed while tracking Anoplophora glabripennis]. However, it is possible that reproductively driven movements may become apparent if the weevils were observed during a different part of the season, or with individuals of a different reproductive status.

There were a number of physical and spatial characteristics that contributed to the weevils’ selection of new host trees. In Study A, more weevils were captured by trees with larger stem diameters, while in Study B, more weevils selected trees that were taller. These results support the findings of Klingenberg et al. (2010b), and give strength to previous studies suggesting that the susceptibility of particular trees to attack by H. warreni is related primarily to tree size. For example, Cerezke (1970) found that stem diameter at stump height (d.s.h.) was a stronger predictor of both percentage of both old and current attacks than altitude, duff depth, tree density, or average stand age. Further, Cerezke (1994) suggested that weevil attack incidence increases with increased tree diameter, while Schroff et al. (2006) and Klingenberg et al. (2010a) found that weevil attack rates and frequency of climbing behavior, respectively, increased with increased tree height. Finally, weevils may not attack very small trees, as Cerezke (1994), working in a 3- to 9-yr-old stand, with tree heights up to 3 m, found the weevils did not attack trees <1.5 m in height. Larger trees represent a better host environment because they provide a more abundant food source, more area of available bark tissue for oviposition (Cerezke 1994), and a better source of shade and shelter. This last benefit may be especially applicable to the weevils’ movements in Study B, as the environmental conditions in this study were likely much drier and more exposed than would considered optimal for the species, and insects in higher temperatures or in areas of less shade may seek host plants primarily for water requirements due to desiccation risk (Papaj and Rausher 1983).

In Study B, there was a strong tendency for individuals of H. warreni to select trees closest to their release point. This may suggest that the weevils do not strongly discriminate between the characteristics of individual trees, and many hosts are potentially suitable. Analogously, Zimmerman (1979) found that bumblebees (Bombus flavifrons), foraging in patches of Polemonium foliosissimum flowers, foraged randomly with respect to direction but typically flew to either the first or second nearest neighbor flower. This suggests that they were selecting flowers based primarily on minimizing flight distances rather than actively selecting for particular flower characteristics.

The results of Study A suggested that weevils avoided climbing trees that had needles discolored either yellow and/or red, indicating that they were dead or dying, most likely killed by prior weevil attack. This contrasts with the findings of Klingenberg et al. (2010b), who found that individuals of H. warreni climbed dead trees as frequently as live trees. However, it is uncertain if the weevils were actually able to detect differences in tree health in this study, or if the differences in results between the two studies are due to differences in methodology, site factors, etc.

There were several significant interaction effects that affected the likelihood of a weevil selecting a particular tree in both studies. In Study A, the regression model included significant negative interactions between diameter and both nearest neighbor distance and APA. In Study B, there was a negative interaction between a given tree’s height and the distance of that tree to its nearest neighbor. These results suggest that smaller trees are even less likely to be selected if they are close to other trees, while being more likely to be selected if they are on their own. These trends may be explained if individuals of H. warreni are more attracted to groups of large trees than to large trees in isolation, but that groups of small trees do not have the same effect. Further, it is possible that smaller trees are relatively more likely to be selected if they are not next to a large tree, as smaller trees may be more apparent to searching weevils if they are in an open area rather than adjacent to and hidden by a dominant larger tree. Although not significant at α = 0.05 (Table 5), the results of Study B also suggested a possible positive interaction between distance from release and tree height on the likelihood an individual of H. warreni selecting that tree. Therefore, the weevil’s preference for taller trees may have increased as the distance increased. This trend may be related to the apparent size of the trees, as perceived by the weevils, as tall trees at large distances can appear similar to small trees at closer distances. However, many species of insects are able to estimate distances to objects by using methods such as the ‘peering’ behavior observed in desert locust nymphs (Schistocerca gregaria), where the insect moves its head side to side and estimates distance by relative movements of the distant object (Wallace 1959), or by visual ‘looming’, where the size of an object increases against a background as the insect moves towards it, such as the looming-sensitive neurons observed in the hawk moth Manduca sexta (Wicklein and Strausfeld 2000). Regardless of whether the weevils are able to effectively estimate distances, the presence of these various interaction effects may suggest that they are primarily identifying potential hosts through the use of vision. Thus, our findings add to the evidence that the weevils search for trees or groups of trees visually based on their silhouettes (Cerezke 1994, Machial et al. 2012a), confirming and extending on the results of Machial et al. (2012a).

We did not observe any overall trends in dispersal directions of H. warreni individuals. There were no consistent trends observed for the movements of H. warreni, either in overall movement directions, turn directions, or correlations between movements and either the closest or largest trees within a given plot in Study B. It is thus unlikely there are any strong general trends or cues that dictate the movement directions or patterns of the species, e.g., such as those which would be present from celestial or solar cues. This pattern is similar to the results observed by Machial et al. (2012b) and Klingenberg (2010b), who both found that individuals of H. warreni moved non-directionally faster in areas devoid of suitable host trees, and to those of Schroff et al. (2006) who found that the spatial distribution of weevil attacks tended to be random rather than clumped. This suggests that perhaps individual weevils search randomly for potential host trees prior to perceiving a possible host, and then use directional movements to move towards a potential host once it has been found. Similar results have been described for other insect species. For example, Laing (1937) found that the chalcid Trichogramma evanescens, which primarily parasitizes lepidopteran eggs, searches randomly when a host is not yet perceived, but once a possible host has been detected, moves towards it using directed movements.

The results of these studies suggest that the movements of H. warreni may be either random or directional based on the weevils’ environment and on scale. Prior studies have suggested that ecological phenomena may be specific to the scale at which they are studied and may have effects of different magnitudes or even reverse at different scales. For example, Saint-Germain et al. (2007) found that certain wood-feeding cerambycid and scolytine species were attracted to volatiles from their host trees at large (patch-level) scales, but not at small (tree-level) scales. In addition, the choice of scale in ecological experiments often does not reflect the scale of the organism, and instead is simply based on convenience or on the work of previous researchers (Wiens 1989). This highlights the importance of further research into the weevils’ movements on scales larger than that of individual tree selection, as movements between patches of potential host trees are critical to determining the migration of H. warreni into newly replanted patches of lodgepole pine (Klingenberg et al. 2010a), which are key to the reforestation process after large-scale harvesting or natural disasters.

## Supplementary Material

ieaa063_suppl_Supplementary_Material
