# Supplementary material for: Random and Directed Movement by Warren Root Collar Weevils (Coleoptera: Curculionidae), Relative to Size and Distance of Host Lodgepole Pine Trees
Source: J Insect Sci. 2020 Jul 24;20(4):9. doi: 10.1093/jisesa/ieaa063 (PMC7380461; doi:10.1093/jisesa/ieaa063)
Supplement: ieaa063_suppl_Supplementary_Material [file ieaa063_suppl_supplementary_material.docx]

# Supplemental Material

## **Study A**

## A.1. Model selection by AIC value, representing the total number of captures of *Hylobius warreni* by funnel traps on lodgepole pine trees.

Table A.1.1. Results of the best-fit linear regression models by Akaike Information Criterion (AIC) value (all models with AIC <164), as well as the null model containing only an intercept, a model with only diameter, and the starting model without interaction terms, used to determine the best model describing the number of *H. warreni* captures during a capture-mark-recapture study in a lodgepole pine stand. D= “Diameter”, NN= “Nearest Neighbour”, APA= “Area Potentially Available”, and C= “Colour”, L= “Leaning”. Variables were centered around the mean, and the dependent variable (total number of captures for each trap was ln(x+1) transformed). For further discussion of the variables considered, see the text.

| Model | AIC |
| --- | --- |
| D+APA+NN+C+L+NN:L+D:APA+D:NN | -164.91 |
| D+APA+NN+C+L+D:APA+D:NN | -164.79 |
| D+APA+NN+C+L+NN:L+APA:C+D:NN+C:L+APA:C | -164.29 |
| D+APA+NN+C+L+NN:L+D:APA+D:NN+APA:NN | -164.28 |
| D+APA+NN+C+L+NN:L+D:APA+D:NN+APA:C | -164.10 |
| D+APA+NN+C+L | -158.39 |
| Intercept and Diameter only | -146.07 |
| Null (Intercept only) | -78.37 |

## **Study B**

## B.1. Schematic diagrams and photographs of study site at the Prince George Tree Improvement Station, used for host location tracking experiments of *Hylobius warreni.*


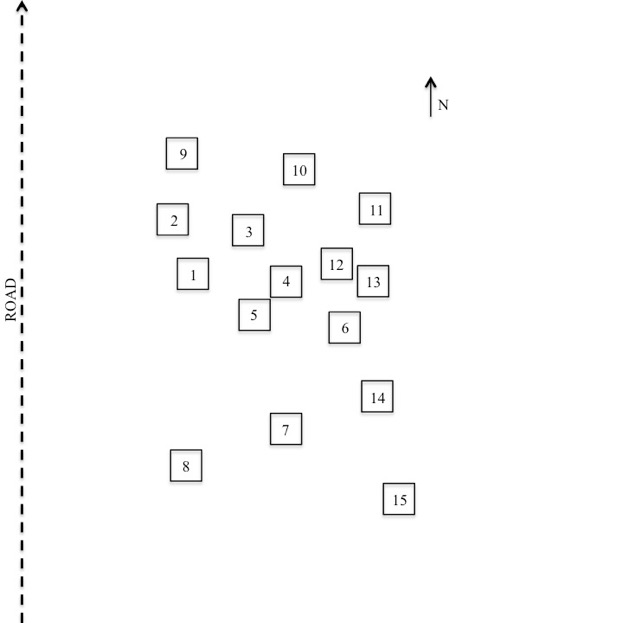


Figure B.1.1. Schematic representation of the positions of 5 m X 5 m plots of trees relative to each other and to the edge of the access road in a lodgepole pine dominated stand at the Prince George Tree Improvement Station, used in a host location harmonic radar tracking experiment of *Hylobius warreni* in the spring and summer of 2013 and 2014.


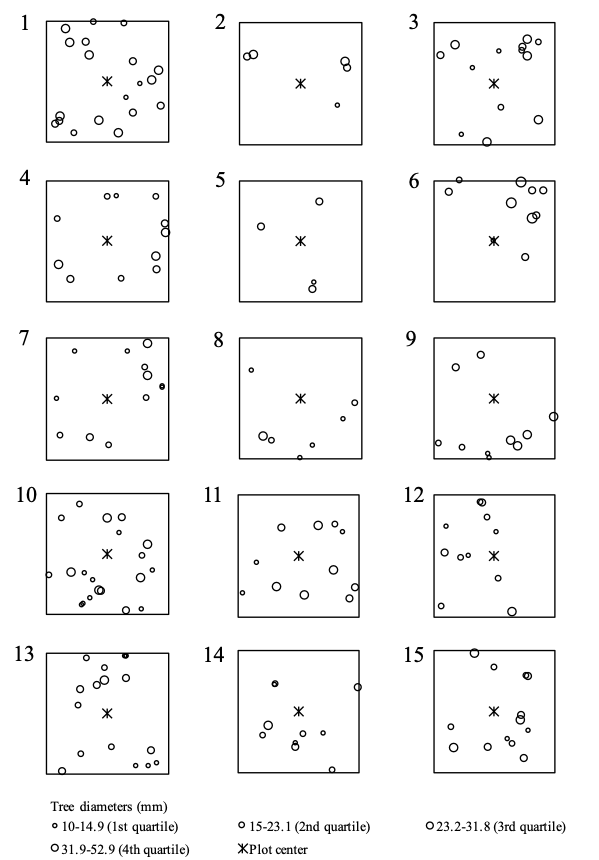


Figure B.1.2. Arrangement of trees in plots numbered 1-15, used for host location harmonic radar tracking experiments in the spring and summer of 2013 and 2014. Plots are 5m X 5m in diameter. Tree sizes not to scale.


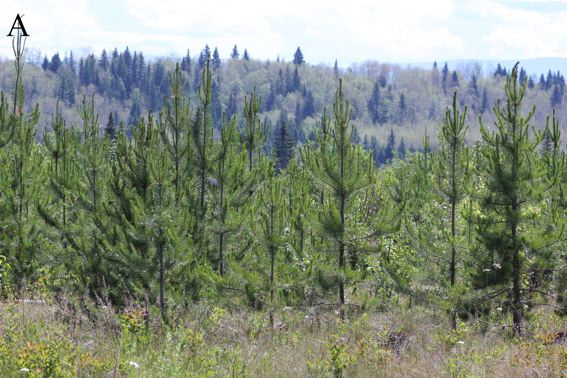


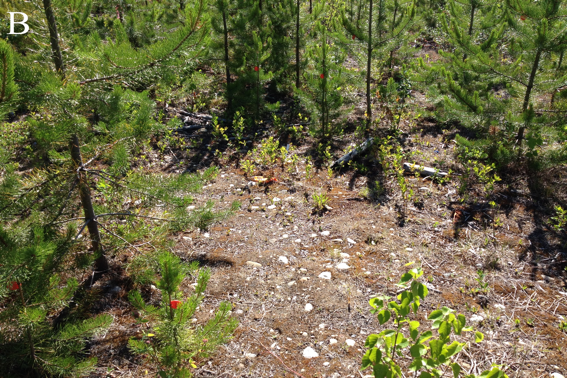


Figure B.1.3. Photographs of site used for host location harmonic radar tracking experiments in the spring and summer of 2013 and 2014. Photo A is taken from the access road, showing the overall site characteristics, while Photo B shows an established plot used for trials, with a marked center (indicated by the arrow above) and tagged trees on all sides. Photos A) BS Lindgren, B) SL Balogh.

## B.2. Model selection by AIC value, representing *Hylobius warreni* selection of trees.

Table B.2.1. Results of mixed effects logistic regression models by Akaike Information Criterion (AIC) value (AIC <120), used to determine weevil selection of trees during host location harmonic radar tracking experiments in the spring and summer of 2013 and 2014. D=Distance of tree from plot center, H= Total tree height, NN=Distance from tree to nearest neighbour tree. The random effect included was Plot, and fixed effect variables were centered around the mean.

| Model | Z_β0_ | P_β0_ | Z_β1D_ | P_β1D_ | Z_β2H_ | P_β2H_ | Z_β3NN_ | P_β3NN_ | Z_β4D*H_ | P_β4D*H_ | Z_β5H*NN_ | P_β5H*NN_ | Z_β6D*NN_ | P_β6D*NN_ | AIC |
| --- | --- | --- | --- | --- | --- | --- | --- | --- | --- | --- | --- | --- | --- | --- | --- |
| Null | -8.27 | <.001 | - | - | - | - | - | - | - | - | - | - | - | - | 143.01 |
| D+H | -6.94 | <.001 | -4.26 | <.001 | 3.05 | 0.002 | - | - | - | - | - | - | - | - | 117.23 |
| D+H+D:H | -6.01 | <.001 | -4.24 | <.001 | 3.33 | <.001 | - | - | 1.99 | 0.05 | - | - | - | - | 115.33 |
| D+H+NN | -6.89 | <.001 | -4.24 | <.001 | 2.98 | 0.003 | -0.80 | 0.42 | - | - | - | - | - | - | 118.54 |
| D+H+NN+D:H | -6.01 | <.001 | -4.20 | <.001 | 3.24 | 0.001 | -0.61 | 0.54 | 1.91 | 0.06 | - | - | - | - | 116.94 |
| D+H+NN+H:NN | -6.73 | <.001 | -4.30 | <.001 | 2.07 | 0.039 | -0.91 | 0.36 | - | - | -2.33 | 0.02 | - | - | 115.67 |
| D+H+NN+D:H+D:NN | -6.01 | <.001 | -4.21 | <.001 | 3.20 | 0.001 | -0.58 | 0.56 | 1.90 | 0.06 | - | - | -0.19 | 0.85 | 118.91 |
| D+H+NN+D:H+H:NN | -6.16 | <.001 | -4.28 | <.001 | 2.59 | 0.010 | -0.41 | 0.68 | 1.82 | 0.07 | -2.27 | 0.02 | - | - | 114.37 |
